# Long-term follow-up and treatment of lamellar hole-associated epiretinal proliferation presenting with exudative perivascular anomalous complex

**DOI:** 10.1016/j.ajoc.2025.102446

**Published:** 2025-09-30

**Authors:** Gy. Dósa, Joanne M. Fuller, Madeleine Zetterberg, Martin Breimer, Lada Kalaboukhova

**Affiliations:** a Region Västra Götaland, Sahlgrenska University Hospital, Department of Ophthalmology, 431 80, Mölndal, Sweden; b Department of Clinical Neuroscience and Physiology, Sahlgrenska Academy, University of Gothenburg, SU/Sahlgrenska, Blå Stråket 7, plan 3, SE-413 45, Sweden

**Keywords:** Exudative perifoveal vascular anomalous complex, Large retinal capillary aneurysm, Lamellar hole-associated epiretinal proliferation, Epiretinal membrane, Ellipsoid zone, Anti-vascular endothelial growth factor, Dexamethasone intravitreal implant

## Abstract

**Purpose:**

To report a patient with lamellar hole-associated epiretinal proliferation (LHEP), presenting with exudative perifoveal vascular anomalous complex (ePVAC), who was initially treated with intravitreal anti-vascular endothelial growth factor (anti-VEGF), aflibercept 2 mg followed by dexamethasone intravitreal implant 0.7 mg.

**Observations:**

A complete ophthalmologic examination was performed on a 74-year-old woman who was referred for unilateral blurred vision. The patient was in good general health with no history of diabetes, hypertension, or blood dyscrasias and was followed up with swept source optical coherence tomography (SS-OCT). The patient was followed from September 2019 to March 2024.

Multimodal imaging of the left eye showed epiretinal proliferation (ERP) with lamellar hole (LM) formation associated with perifoveal isolated large aneurysmal change, accompanied by small hemorrhage, intraretinal exudation and small hard exudate accumulations.

The patient received three monthly intravitreal injections as a loading dose (LD), followed by treat and extend regimen. Four weeks post LD the mean central retinal thickness (CMT) decreased from 467 to 288 μm, LHEP cystoid spaces were reduced, and best corrected visual acuity (BCVA) improved from 20/120 to 20/60. Treatment continued with extended intervals. After 11 injections there was a follow-up loss at 18 weeks with deterioration of BCVA.

After a total of 18 aflibercept injections it was decided to switch to dexamethasone intravitreal implant. At three months there was anatomical improvement and the ePVAC lesions almost disappeared. At 14 weeks after the second implant, OCT confirmed further anatomical improvement and involution of the PVAC lesions. At 10 weeks after the third dexamethasone intravitreal implant there was a deterioration due to the progression of cataract and BCVA had dropped to 20/120. The intraocular pressure was between normal limits during the follow-up period.

**Conclusions and importance:**

Anti-VEGF therapy is less effective than dexamethasone intravitreal implant with a treatment interval greater than 12 weeks.

## 1 Claim of priority

After conducting a literature review on (February 20, 2025) utilizing PubMed, Google Scholar, CINAHL, and SCOPUS using the key words (lamellar hole associated epiretinal proliferation and exudative perifoveal anomalous complex and intravitreal dexamethasone implant), we did not find any prior reports of lamellar-hole-associated epiretinal proliferation (LHEP) presenting with exudative perifoveal anomalous complex successfully treated with intravitreal dexamethasone implant with a longer follow-up.

In this case report we describe a patient with this very rare clinical entity treated successfully with intravitreal dexamethasone implant after switching from long-term intravitreal anti–VEGF treatment (aflibercept, 2 mg). We have found a better and longer effect with dexamethasone intravitreal implant.

This option could be the first line treatment in patients who have undergone complication-free cataract surgery with intact posterior capsule and in patients with no advanced glaucomatous optic disc damage.

## 2 Introduction

Exudative perivascular anomalous complex (ePVAC) is a retinal capillary abnormality first described as perifoveal exudative vascular anomalous complex (PEVAC) by Querques et al.^1^ in 2011 as an isolated, unilateral, usually unifocal, large perifoveal aneurysmal lesion associated with surrounding microvascular changes in an otherwise healthy patient. However, perifoveal vascular anomalous complex (PVAC) could be associated with other concomitant ocular disorders including age related macular degeneration (AMD) and pathologic myopia.^2^^,^^3^ Sacconi and colleagues expanded the spectrum of this vascular abnormality reporting the multimodal imaging features in a series of 15 patients.^2^

Recently, two forms of this condition have been differentiated between a non-exudative (nePVAC) and an exudative (ePVAC), the first being the preclinical stage of the latter.^3^ While nePVAC lesions are asymptomatic in the initial phase, patients with ePVAC typically complain of visual decline and metamorphopsia caused by intraretinal exudation associated with the aneurysmal lesion.

In optical coherence tomography (OCT), ePVAC is characterized by a unilateral, round, hyperreflective isolated lesion often accompanied by intraretinal cystoid spaces without neovascularization. The Delphi consensus study of international retinal specialists defined this specific OCT lesion as large retinal capillary aneurysm (LRCA) with at least 100 μm in diameter or greater on OCT, with a characteristic hyperreflective aneurysmal wall and hyporeflective lumen.^4^

Fluorescein angiography (FA) and indocyanine green angiography (ICGA) show no associated retinal or choroidal vascular abnormalities.^1^

PVAC lesions should be differentiated mainly from macroaneursym/large microaneurysm, type 1 macular telangiectasia (MacTel 1) and “nascent” type 3 lesions.^5^ Macroaneurysms and microaneurysms are usually caused by other retinal vascular disorders such as retinal vein occlusion, diabetic retinopathy, and inflammatory diseases. The terminology of retinal capillary macroaneurysms has been proposed in 2019 by Spaide and Barquet for large, solitary, and persistent aneurysms greater than 200 μm, which arise from capillaries and grow over time. Nevertheless, the definition of retinal capillary macroaneurysms can include a wider spectrum, as they are potentially not limited to the area around the fovea.^6^ MacTel 1, especially subtype 1B, typically presents in young patients as focal exudative telangiectasia, limited to two clock hours in the juxtafoveal area, with intraretinal microangiopathy, affecting both the superficial and deep capillary plexus. Contrastingly ePVAC affects older patients with well-defined and isolated aneurysmal lesion without other telangiectatic capillaries. The capillary rarefaction in the non-exudative form of PVAC, confirmed with fluorescein angiography and OCTA-angiography is considered as the hallmark of the PVAC spectrum.^3^^,^^7^ In contrast to MacTel 1, ePVAC did not usually respond to anti-VEGF therapy.^8^ Differentiation from the earliest form of type 3 macular neovascularization (NMV), namely “nascent type 3 “can be challenging, especially with respect to nePVAC. This type 3 of NMV is characterized by intraretinal hyperreflective foci with detectable flow using OCT-A in the deep vascular complex, without any evidence of exudation or mild microcystic change until the lesion progresses to involve the retinal pigment epithelium (RPE) and sub-RPE space.^9^ However, these stage 1 lesions are almost always associated with downward growth toward the RPE with progressive exudation (stage 2 and 3).^9^ Unlike ePVAC type 3 neovascularization responds promptly to anti-VEGF therapy.^10^^,^^11^

DRAMA or deep retinal age-related microvascular anomalies, described by Cabral et al. in 2022 have overlaps with ePVAC and these lesions could be the part of the same spectrum.^12^ These lesions have two subtypes, the first being small diameter perifoveal capillary dilatations with hyperreflective walls within the inner nuclear layer, the second is characterized by multiple, vascular dilatations extending posteriorly into Henle fiber layer, with similar reflectivity to adjacent normal retinal capillaries.

Lamellar hole–associated epiretinal proliferation (LHEP), first described by Witkin et al.^7^ as thickened epiretinal membrane (ERM) and later as LHEP by Pang et al.^10^ in 2014, is epiretinal tissue with homogenous moderate reflectivity that is visible through optical coherence tomography (OCT) in patients with lamellar macular holes (LMHs) or full-thickness macular holes (FTMHs). LHEP was found to originate from the proliferation of Müller cells onto the epiretinal surface.^9^^,^^10^

Siedlecki et al. described the first case of a patient with lamellar-hole associated epiretinal proliferation (LHEP), presenting with PEVAC and suboptimal response to anti-VEGF therapy.^11^

Here, we describe the long term (55 months) follow-up and treatment of a patient with exudative PVAC seemingly communicating with edematous LHEP found above a lamellar macular hole. Initially, the patient was treated with intravitreal anti-vascular endothelial growth factor (VEGF) injections, aflibercept 2 mg, but later the regimen was changed to dexamethasone intravitreal implant according to Epstein protocol.^13^

## 3 Case report

A 74-year-old woman presented at our department with decreased vision in the left eye. She complained of worsening symptoms for 13 months without significant metamorphopsia. Medical history was negative for diabetes, hypertension, and blood dyscrasias.

The best corrected visual acuity (BCVA) of the patient was 20/20 and 20/120 in the right and left eye respectively. In both eyes, anterior segment examination was unremarkable, except for mild corticonuclear cataract formation. Intraocular pressure was normal. On fundus examination an isolated aneurysmal lesion was observed inferotemporal to the fovea of the left eye with accumulation of small hard exudates both nasally and temporally of the fovea and with a dot hemorrhage in the temporal macula.

**Fig. 1:**
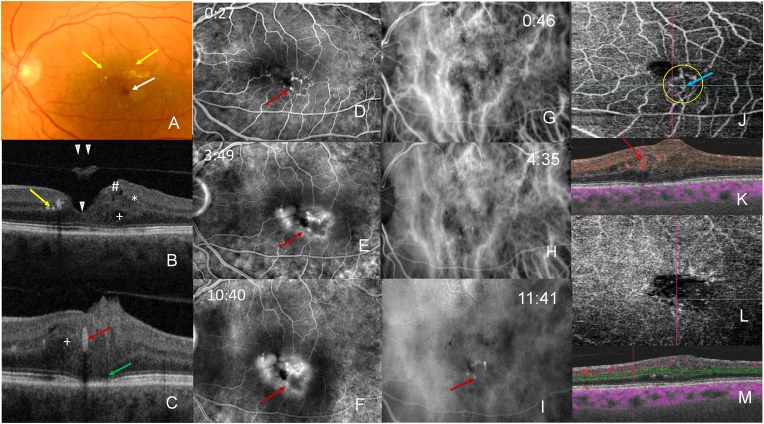
Multimodal imaging of exudative perifoveal anomalous vascular complex (ePVAC) and lamellar hole-associated epiretinal proliferation (LHEP). (A) Color fundus photography revealed dot hemorrhage (white arrow) and hard exudates (yellow arrows). SS-OCT B scan (B–C) showed a lamellar macular hole (arrowhead ∇), posterior vitreous detachment with operculum (double arrowhead ∇∇), a single oval lesion with background shadowing (red arrow) highly suggestive of ePVAC, hyperreflective foci corresponding to the hard exudates (yellow arrow). The ellipsoid zone (EZ) was disrupted (green arrow), intraretinal cavities (plus sign +) and LHEP (hash #) with intra-LHEP fluid (star ∗). Vessel imaging: (D) FA revealed a well-defined hyperfluorescent lesion in the early phase (red arrow) with leakage in the mid- and late phase (E, F). (G–I) ICGA demonstrated an anomalous vascular network (red arrow) with no leakage. (J–M) OCT-A, superficial (J) and deep capillary plexus (L) and corresponding cross-sectional images (K, M) disclosed one lesion with blood flow (red arrow). An area of capillary rarefaction (within the yellow circle) and microvascular dilatations (turquoise arrow) could be detected in the superficial capillary plexus slab (J).

Multimodal imaging included fundus photography (CX-1 Hybrid Digital Mydriatic/Non-Mydriatic Retinal Camera, Canon Medical Systems USA), swept source optical coherence tomography (SS-OCT, DRI-OCT Triton Topcon, Tokyo, Japan), fluorescein (FA)/indocyanine green angiography (ICGA) (Topcon TRC 50 Ex Fundus Camera for angiography, Japan) and OCT angiography (OCTA, Topcon SS OCT- Angio, Tokyo, Japan). Fundus photography demonstrated a red dot-like lesion (Fig. 1, A, white arrow) and accumulation of hard exudates in the perifoveal area of the left eye (Fig. 1, A, yellow arrows).

Whereas OCT showed no retinal pathology except for a classic, thin epiretinal membrane in the right eye, the left eye featured posterior vitreous detachment with an operculum (Fig. 1, B, double arrowhead ∇∇). A significant amount of LHEP (Fig. 1, B, hash #) covered the temporal fovea, containing irregularly formed hyporeflective cystoid spaces (Fig. 1, B, plus sign +), a single oval lesion measuring 139 × 130 μm with a hyperreflective wall and a lumen with background shadowing (Fig. 1, C, red arrow) and hyperreflective foci corresponding to the hard exudates (Fig. 1, B, yellow arrow). The ellipsoid zone (EZ) was disrupted (Fig. 1, C, green arrow).

Both FA and ICGA demonstrated an anomalous vascular network, that clearly presented with intraretinal and intra-LHEP exudation (Fig. 1, D–F, G-I). The hyperfluorescent structures correlated with a dilated anomalous vascular network. OCT angiography revealed that the abnormal vessels extended between the superficial and deep retinal plexus (Fig. 1, J–M), suggesting the diagnosis of an ePVAC. The ePVAC additionally presented with an area of capillary rarefaction that could be detected in the superficial and deep capillary plexus slab (Fig. 1, J, yellow circle) accompanied by microvascular dilatations that could be seen temporal to the fovea (Fig. 1, J, turquoise arrow). No flow was detected inside the LHEP by OCT-A.

**Fig. 2:**
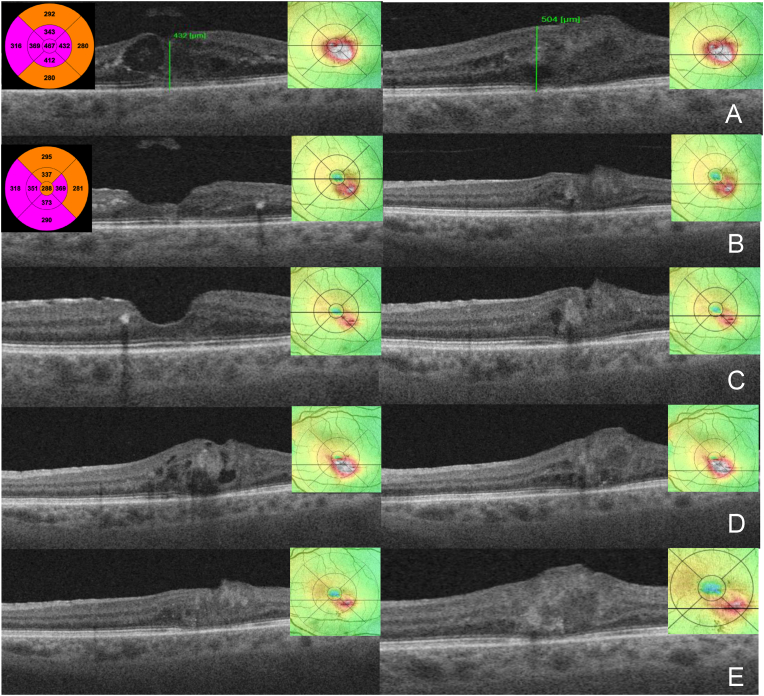
(A–E) SS-OCT B scans and ETDRS area maps through and beneath the fovea (A–C) respectively at two different distances beneath the fovea (D, E) during the follow up and treatment with aflibercept 2 mg (“E”) in the first and second year (A) Before treatment initiation the mean central retinal thickness (CRT) on macular thickness map 467 μm, central foveal thickness 432 μm, retinal thickness (RT) 504 μm manually measured through the capillary macronaurysm. (B) 4 weeks after E3, (C) 11 weeks after E6, (D) 18 weeks after E10, another ePVAC lesion has appeared, (E) 4 weeks after E11.

**Fig. 3:**
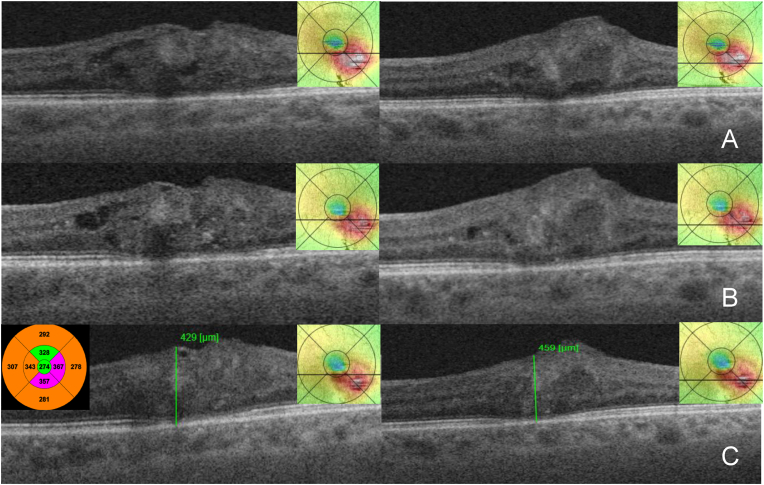
SS-OCT B scans and ETDRS maps during follow up and treatment in the third and fourth year, B scans at two different distances beneath the fovea. (A) 13 weeks after E17; (B) 9 weeks after E18; (C) 20 weeks after E19, switch to dexamethasone (Oz) intravitreal implant, on macular thickness map CRT 274 μm, RT manually measured through the capillary macroaneurysms 429 and 459 μm respectively.

Given the lack of a tractional epiretinal membrane, we did not recommend macular surgery with internal limiting membrane peeling. After four months, visual function remained unchanged. However, a significant increase of intra-LHEP edema with increased macular thickness was detected, and therefore, anti-vascular endothelial growth factor (VEGF) intravitreal therapy was recommended with treat and extend regimen. Following three intravitreal aflibercept injections every four weeks as a loading dose, OCT revealed a decrease of the mean central retinal thickness (CMT) from 467 to 288 μm (Fig. 2, A and B) and a reduction of LHEP cystoid spaces as well as improvement in BCVA to 20/60. We continued the treatment with 9, 10, 9, 8, 7-week extended intervals. After a total of 11 injections (Fig. 2, C) there was a follow-up loss 18 weeks with deterioration of BCVA. OCT imaging showed an increase of intraretinal fluid and yet another large capillary aneurysm inferior to fovea measuring 149 × 119 μm in addition to the previously known aneurysm (Fig. 2, D). After three monthly aflibercept injections we observed anatomical and functional improvement with BCVA increasing to 20/40 (Fig. 2, E). During further follow-up and treatment with 6, 5, 6, 8, 13-week extended intervals we observed a gradual resolution of intraretinal cystoid spaces and even involution of the PVAC lesions at 20 weeks after the last aflibercept injection (Fig. 3, A and B).

**Fig. 4:**
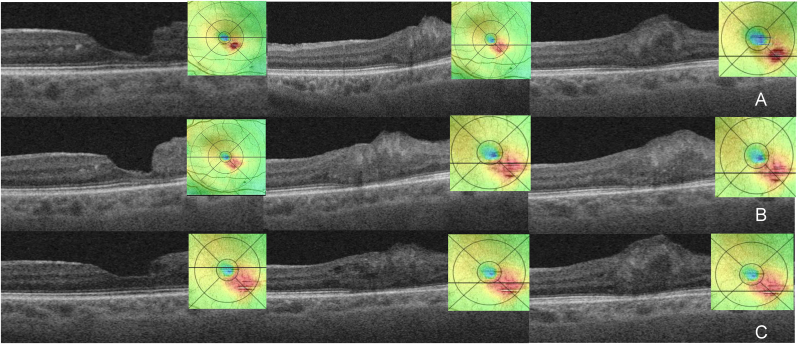
SS-OCT B scans and ETDRS maps during follow up and treatment with dexamethasone (Oz) intravitreal implant, B scans through the fovea and at two different distances beneath the fovea. (A) 13 weeks after Oz 1; (B) 14 weeks after Oz 2, (C) 15 weeks after Oz 3.

We switched to 0.7 mg dexamethasone intravitreal *implant* (Fig. 3, C). At 13 weeks after the intravitreal dexamethasone implant there was anatomical improvement and the PVAC lesions had almost disappeared (Fig. 4, A). The patient has been scheduled for the next dexamethasone implant at 12 weeks. At 14 weeks after the second dexamethasone implant, OCT confirmed further anatomical improvement and involution of the PVAC lesions (Fig. 4, B). At 10 weeks after the third dexamethasone implant there was a deterioration due to the progression of cataract and BCVA had dropped to 20/120 (Fig. 4, C). The intraocular pressure was between normal limits during the follow-up period.

## 4 Discussion

Our case showed significant regression of the original large retinal capillary aneurysms and improvement in exudation in response to intravitreal injections of anti-VEGF followed by dexamethasone intravitreal implant.

Although multimodal imaging has provided a better understanding of pathophysiological mechanisms and treatment outcomes of different retinal diseases, the exact pathogenesis of exudative PVAC lesions is still unclear.

Different hypotheses have been proposed. The hypothetical mechanism of focal or progressive endothelial cell degeneration in patients with PVAC, without other retinal/choroidal vascular or inflammatory diseases, was proposed by Querques et al. since its first description in 2011.^1^^,^^2^ Hence, increased endothelial damage may lead to exudation and progression from nePVAC to ePVAC.^3^ Both perifoveal aneurysm and perilesional capillaries rarefaction seem to result from pericyte loss, from breakdown of pericytes’ basement membrane by matrix metalloproteinase 9.^1^

It was hypothesized by Spaide and Barquet that in larger isolated aneurysms, derived from capillaries in the macular area, there is an increased expression of matrix metalloproteinase 9, which functions to breakdown basement membrane proteins and accounts for a proportionate drop in pericyte coverage.^5^

The study by Lopez-Luppo et al.^14^ suggests that matrix-metalloproteinase 9 may work to decrease the structural integrity of the basement membrane, potentially leaving less resistance to aneurysmal expansion. By Laplace's law,^15^ the wall tension for an aneurysm increases proportionately to its radius. Thus, there may be two factors facilitating enlargement; decreasing wall strength and increasing wall tension as the lesion grows larger, both of which may lead to expansion of the aneurysm. These expansionary factors may also augment leakage from the microaneurysm in ways that are not necessarily VEGF-dependent.

Smid et al. hypothesized that aging is a critical factor in the development of PVAC lesions, given that an association between microvascular rarefaction and aging has already been established.^16^, ^17^, ^18^, ^19^ Siedlecki et al. hypothesized a glial cell–driven genesis, secondary to Müller cells necrosis in explaining their case of concomitant ePVAC and lamellar hole-associated epiretinal proliferation.^11^ The first possible etiologic scenario described by the authors might be the coincident presence of a lamellar hole with associated epiretinal proliferation and ePVAC, which gained connection to LHEP tissue because of long-standing disease and LHEP migration towards the vascular lesion. The second possible etiologic scenario may be that Müller cell dropout in deep retinal layers has led to vascular destabilization, generating the telangiectatic vessels demonstrated on OCT angiography.^10^ Necrosis of Müller cells may lead to cystoid spaces.^16^^,^^17^

LHEP has a yellow color, as it is removed from the macula during vitrectomy. Shiraga et al. concluded that this thickened epiretinal tissue contained luteal pigment.^20^ Choi et al. confirmed that the optical density of LHEP was such that it seemed to connect to the outer plexiform and other middle layers of the retina before extending over the ILM on the retinal surface.^21^ The outer plexiform layer of the fovea contains the highest density of lutein and zeaxanthin.^21^^,^^22^ Whether the pigment diffuses into the LHEP or expands onto the retina with proliferation of the tissue is uncertain.^20^^,^^21^

Long term follow-up and treatment of our patient suggest the role of a VEGF mediated as well as a low grade focal inflammatory process in the pathophysiology of ePVAC. Besides these, the coincident presence of LHEP refer to a possible Müller cell driven pathogenesis in this vascular abnormality.

In addition, ePVAC could be a response to anatomical variation, particularly to a prepapillary arterio-arterial loop, in chronic slow flow retinopathy.^23^

Lesions occurring in ePVAC disease are usually not progressive and cause a moderate impact on vision, with visual decline and metamorphopsia caused by intraretinal exudation associated with the large retinal capillary aneurysmal lesions.

Sacconi et al. and Kim et al. reported a spontaneous improvement in the intraretinal cystoid spaces associated with an ePVAC lesion in two patients.^2^, ^24^ Moreover, Verhoekx et al. reported seven patients with spontaneous resolution of intraretinal cystic spaces during follow-up.^18^ The authors suggested that there can be a spontaneous resolution of intraretinal cystic spaces because of the natural history of the disease, rather than the effect of the anti-VEGF injections. In three patients, they even observed a complete disappearance of the ePVAC lesion.

Due to the rarity of this clinical entity, the appropriate management of exudation secondary to PVAC has not been established.

Exudative PVAC is usually unresponsive to intravitreal anti-VEGF injection. Mrejen et al. reported the resolution of intraretinal cystic spaces after 13 anti-VEGF injections (two injections of aflibercept and 11 injections of ranibizumab) in one patient.^6^ However, as the improvement did not occur initially but after several months of follow-up, the possibility of natural improvement in this patient rather than the anti-VEGF injections could not be ruled out.

Focal thermal laser photocoagulation has also been described as an effective treatment. Photocoagulation produces coagulative necrosis of cells and coagulation of blood contained in the aneurysm, which may lead to involution of the lesion and resolution of leakage.^6^ Subthreshold micropulse laser, another form of thermal laser photocoagulation is a potential treatment of choice for ePVAC as stand-alone intervention or following anti-VEGF injections.

Long-term outcome data with subthreshold focal laser treatment as noninvasive modality in ePVAC is not available yet.

Tombolini et al.^25^ presented a case of a patient with ePVAC who was treated with topical non-steroidal anti-inflammatory drug (NSAID). Given the fact that there is no definitive evidence proving beyond any doubt the role of diclofenac in reducing macular edema, including exudation from PVAC, there is clearly a possibility that the reduction in intraretinal exudation in their case was not related to any therapeutic effect of diclofenac, but rather occurred as a coincident event.

Although ePVAC was initially described to be present only in an otherwise healthy individual with normal eyes, newer associations of this entity have been reported in the literature. Sacconi et al. evaluated 15 eyes with ePVAC and defined an expanded spectrum of the association including AMD and myopic macular degeneration.^2^ Fernández-Vigo et al. have described an atypical case of ePVAC with bilateral presentation and multifocal lesions in one eye.^26^ Recently, it has also been shown that diabetic patients can demonstrate these lesions in the presence or absence of diabetic retinopathy changes.^27^^,^^28^ Furthermore, Nataraj et al. presented a patient with refractory perifoveal exudative vascular anomalous complex-like lesion in one eye, who had concurrent bilateral mild non-proliferative diabetic retinopathy (NPDR) responding to intravitreal dexamethasone implant.^29^

While the inciting events for these pathological changes are uncertain, we suppose that a local, potential low-grade inflammation could be one possibility.

The exact mechanism for dexamethasone response in exudative PVAC lesions remains unclear, but possible anti-permeability, anti-inflammatory and anti-angiogenic mechanisms are plausible.

The primary therapeutic actions of intravitreal corticosteroid implants are stabilization of the blood-retinal barrier (BRB), resorption of fluid and protein leakage (exudation), and down-regulation of the underlying inflammatory stimuli.

At the BRB, corticosteroids act to maintain tight junction integrity at the level of endothelial/epithelial cell border. They reduce the vascular permeability through their effects on transcellular aquaporin-4 (AQP4) and potassium channels, the two main channels controlling retinal fluid movement in retinal Müller cells.^30^

The potential role of inflammatory markers in ePVAC lesions needs to be further explored by evaluating the serum and vitreous samples of these patients who are usually otherwise healthy systemically.

An intravitreal dexamethasone implant can be an alternative treatment for ePVAC when combined anti-VEGF injections and subthreshold micropulse laser therapy are insufficient. However, this treatment option carries significant risks, including accelerated cataract development as in this case and increased intraocular pressure, which can lead to glaucoma.

There is no consensus on standard of care treatment of ePVAC, and more data on relative efficacy of the available treatment modalities are necessary to guide clinicians in their choice of appropriate treatment.

## 5 Conclusion

In conclusion, further research is needed to better elucidate the physiopathological mechanisms to gain better insight and evidence into its mechanism of action and the potential role of a local inflammatory process in the pathogenesis of exudative PVAC lesions.

Clinicians should be aware of the relatively poor prognosis or long recovery time of ePVAC.

## CRediT authorship contribution statement

**Gy. Dósa:** Writing – review & editing, Writing – original draft, Visualization, Supervision, Software, Resources, Project administration, Methodology, Investigation, Formal analysis, Data curation, Conceptualization. **Joanne M. Fuller:** Writing – review & editing, Project administration, Methodology. **Madeleine Zetterberg:** Writing – review & editing, Supervision, Project administration, Methodology. **Martin Breimer:** Writing – review & editing, Visualization, Resources, Project administration, Methodology, Investigation, Formal analysis, Data curation, Conceptualization. **Lada Kalaboukhova:** Writing – review & editing, Supervision, Project administration, Methodology.

## Patient consent

Consent to publish this case report has been obtained from the patient in writing.

## Acknowledgements and disclosures

No funding or grant support.

## Authorship

All authors attest that they meet the current ICMJE criteria for Authorship.

## Declaration of competing interest

None to declare.
